# Immune dysfunction following severe trauma: A systems failure from the central nervous system to mitochondria

**DOI:** 10.3389/fmed.2022.968453

**Published:** 2022-08-30

**Authors:** Geoffrey P. Dobson, Jodie L. Morris, Hayley L. Letson

**Affiliations:** Heart and Trauma Research Laboratory, College of Medicine and Dentistry, James Cook University, Townsville, QLD, Australia

**Keywords:** trauma, hemorrhage, immune, inflammation, mitochondria, system, ALM, cytokines

## Abstract

When a traumatic injury exceeds the body’s internal tolerances, the innate immune and inflammatory systems are rapidly activated, and if not contained early, increase morbidity and mortality. Early deaths after hospital admission are mostly from central nervous system (CNS) trauma, hemorrhage and circulatory collapse (30%), and later deaths from hyperinflammation, immunosuppression, infection, sepsis, acute respiratory distress, and multiple organ failure (20%). The molecular drivers of secondary injury include damage associated molecular patterns (DAMPs), pathogen associated molecular patterns (PAMPs) and other immune-modifying agents that activate the hypothalamic-pituitary-adrenal (HPA) axis and sympathetic stress response. Despite a number of drugs targeting specific anti-inflammatory and immune pathways showing promise in animal models, the majority have failed to translate. Reasons for failure include difficulty to replicate the heterogeneity of humans, poorly designed trials, inappropriate use of specific pathogen-free (SPF) animals, ignoring sex-specific differences, and the flawed practice of single-nodal targeting. Systems interconnectedness is a major overlooked factor. We argue that if the CNS is protected early after major trauma and control of cardiovascular function is maintained, the endothelial-glycocalyx will be protected, sufficient oxygen will be delivered, mitochondrial energetics will be maintained, inflammation will be resolved and immune dysfunction will be minimized. The current challenge is to develop new systems-based drugs that target the CNS coupling of whole-body function.

## Introduction

**FIGURE 1:**
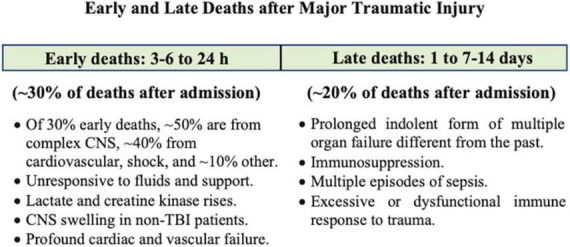
After hospital admission, complications occur at different times and depend upon type and severity of injury. These have been characterized as early and late Deaths. Modified after Rauf et al. (3) and Brohi et al. (4). Patients who died at the point-of-injury or in the first few hours upon arrival to hospital have not been included. CNS, central nervous system; TBI, traumatic brain injury.

Globally, over one billion people sustain traumatic injuries, and over six million die annually (1). Mortality is twofold higher in low- and middle-income countries compared to high-income countries, and up to 5-times higher in resource-limited rural and remote regions (1, 2). In patients who survive the first few hours of hospital admission, complications can occur at different times (Figure 1) (3). The first window is 3–6 to 24 h where CNS dysfunction (∼50% of early deaths) and circulatory collapse (usually manifesting as shock) lead to early deaths (40% of early deaths) (Figure 1) (4–6). The second window occurs over the next few weeks and typically involves infectious complications with a prolonged indolent form of multiple organ failure, immunosuppression and sepsis, referred to as Persistent Inflammation, Immunosuppression and Catabolism Syndrome (PIICS) (∼20% of deaths) (Figure 1) (4, 7, 8). Sepsis develops in ∼10% of these patients and multiple organ dysfunction syndrome (MODS) in around 70% (3, 9, 10). Despite decades of research, little progress has been made in the development of effective drugs to treat the polytrauma patient (2, 11, 12). The lack of progress in drug development may reflect the way we think about the problem (13, 14). In this review, our aim is to discuss the inflammatory and immune mechanisms that are believed to be responsible for early and late secondary injuries and death following traumatic injury, and possible ways to reduce morbidity and mortality from a systems-based perspective. Before doing so, we will briefly discuss the physiological importance of the system.

## Challenging the steady-state and evolutionary internal tolerances

After a traumatic injury, defined as one or more sudden injuries requiring immediate medical attention, the body activates a series of defense mechanisms to restore homeostatic balance. The concept of homeostatic balance was introduced into medicine in 1916 by Cannon (15). Cannon’s genius was to combine the ideas of Pfluger’s “*natural adjustments”* (1877), Bernard’s concept of “*milieu intérieur*” (1878), and Richet’s “*living beings were stable but modifiable*” (1900) into a unified scheme (16). Cannon proposed that every living organism was in a *dynamic state of constancy*, with its constituent parts and processes being *actively* maintained in balance despite external fluctuations (15). The system is not an equilibrium system as it requires a continual flow of matter, energy and exchange with the environment (16, 17). In the mid-1930s, Cannon’s concept was refined to include negative and positive feedback circuits (18), and the system’s steady-state was now viewed as the net sum of negative and positive feedback mechanisms that operate within a range of tolerances, which differ from person to person, and from species to species. The system has evolved such that small injury perturbations are self-limiting and quickly resolved. However, when the trauma overwhelms the system, it triggers a CNS stress response that typically involves excessive sympathetic and neuroendocrine outflows from the brain’s central control, hyperinflammation, immune dysregulation, coagulopathy, endothelial activation and metabolic dysfunction (13, 19, 20). *If homeostatic balance is not restored early, secondary injury processes will amplify and may become life-threatening* (14).

## First line-of-defense: The innate immune system

When I first put forward the biological theory of inflammation 8 years ago, I expressed the idea that this reaction is affected by the intermediation of a physiological continuity between “the cells of the connective tissue, those of the endothelial wall and the leucocytes, which form a complete chain and play the principal part in the inflammation of vertebrates.” The connective tissue cells which are first attacked, would, I thought, transmit the action to the vascular wall, the cells of which would contract to facilitate the passage of the white corpuscles.

Metchnikoff (21) p. 191.

**FIGURE 2:**
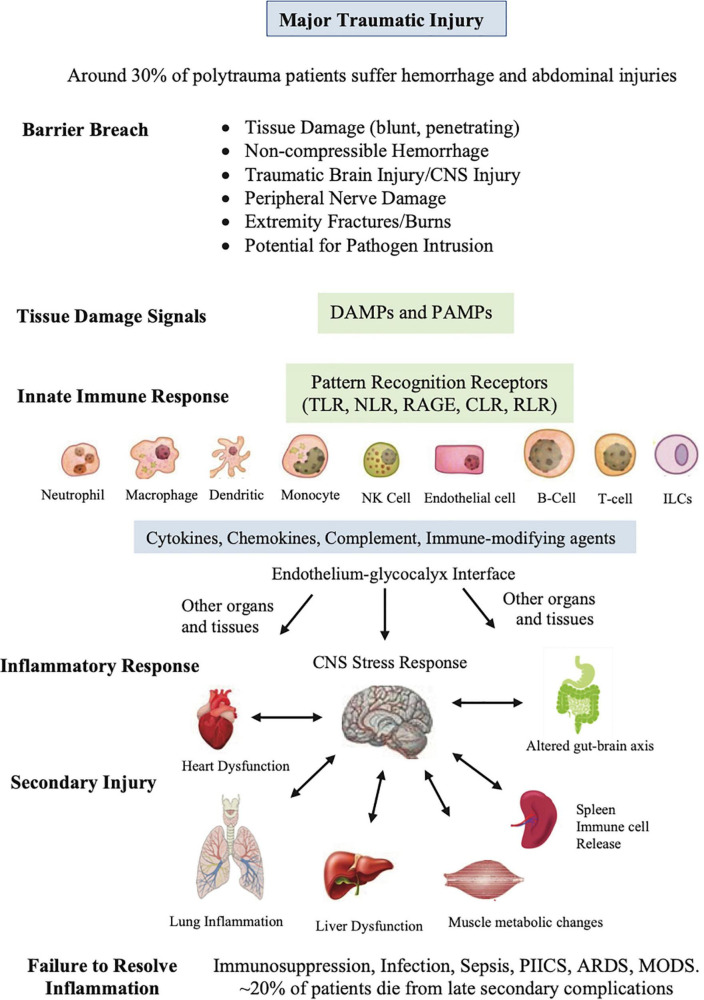
Sequence of events that occur after major traumatic injury. This diverse group of innate cells resident in the tissues detect and respond to changes in the local environment, including damage associated molecular patterns (DAMPs), pathogen associated molecular patterns (PAMPs), neural signals, and other immune-modifying triggers. The pattern recognition receptors on these cells include Toll-like receptors (TLRs), C-type lectin receptors (CLRs), nucleotide-binding oligomerization domain (NOD)-like receptors (NLRs), retinoic-acid-inducible gene-I (RIG-I)-like receptors (RLRs), and receptor for advanced glycation end products (RAGE). PAMPs can be derived from viruses, opportunistic bacteria, fungi, and protozoa and helminths. The innate cells orchestrate an immune response to response to the barrier breach by releasing different inflammatory factors. If dysregulated, the response can lead to secondary injury to the CNS and major organs of the body. The spleen has been included as it is reservoir of platelets, peripheral macrophages, undifferentiated monocytes and other immune cells. ILCs, innate lymphoid cells; NK, natural killer cells, PIICS; Persistent Inflammation, Immunosuppression and Catabolism Syndrome, ARDS; acute respiratory distress syndrome, MODS; multiple organ dysfunction.

Any trauma to the body inflicts a barrier breach in three-dimensional space and one in time. Damage signals from cellular, vascular and nerve injury are sent around the body, and to the CNS *via* nerve afferents and resident damage control mechanisms to begin the process of tissue repair and remodeling (14, 22). Recovery begins by rapidly closing the breach, activating immuno-inflammatory processes, removing damaged cells and killing any invading microbes (Figure 2). Immune defense occurs in two parts: First, there is a local frontline defense from patrolling resident immune cells in tissues, and second, from deployment of additional leukocyte subsets from the circulation. Early defense includes activation of tissue resident macrophages, dendritic cells (DCs), neutrophils (PMNs), mast cells, a subset of memory B cells, natural killer (NK) cells, complement (22–24) and recently characterized resident T cells, referred to as innate lymphoid cells (ILCs), which are believed to interact with other resident cells, and trigger the early adaptive immune response and recruitment of cells from the circulation to repair and restore tissue function (25–29) (Figure 2).

This diverse group of resident innate cells have evolved different pattern-recognition receptors that detect and respond to changes in the local environment, including damage associated molecular patterns (DAMPS), pathogen associated molecular patterns (PAMPs) and other immune-modifying triggers (Figure 2) (30–32). DAMPs are released from damaged, stressed or dying cells, including extracellular and cell membrane, cytosolic, cytoskeleton, nuclear mitochondrial, endothelial and blood components (30, 33), while PAMPs are signature proteins, lipoproteins, nucleic acids and saccharides located on the cell surface or released from invading pathogens. Together, they activate the body’s early immune and inflammatory systems to dial in the right response to repair and restore function (Figure 2). Early post-traumatic DAMP markers include high mobility group box protein 1 (HMGB1), mitochondrial DNA (mtDNA), S100, cell fragments, and many other molecules from injured or dying cells and proteoglycans and glycoproteins from endothelial-glycocalyx shedding (34). Importantly, DAMPs and PAMPs are not mutually exclusive and may share co-receptors and accessory molecules, and form partnerships to coordinate the right response (35).

A 2011 landmark study of Xiao and collaborators shed light on the early activation patterns of the immune system following severe blunt trauma and *burn injuries*. The group reported there was ∼80% activation of the leukocyte transcriptome in the circulation, which they termed a genomic storm (36). This storm developed within 4–12 h and lasted days to weeks. Importantly, in Xiao’s study, what separated patients who developed secondary complications was not the magnitude of the storm, rather the time to resolve it (36). Prolonged resolution times led to worse outcomes. Moreover, both pro-inflammatory and anti-inflammatory pathways were activated early, which challenges the older two-hit and other sequential pro-inflammatory and compensatory anti-inflammatory models of trauma (37). On a cautionary note, although transcriptomic analysis establishes early temporal patterns of change, it provides little or no knowledge into the molecular mechanisms. Future studies should include proteomic and pathway-level analysis to establish the different roles of the early innate (and adaptive systems) to amplify inflammation after severe trauma.

## Early drivers of inflammation and immune dysfunction

Inflammation is universal, beneficial and restorative. However, after major trauma, it can be lethal. As mentioned earlier, the massive release of DAMPs can overwhelm the system and trigger a hyperinflammatory state that, if not resolved in a timely manner, can lead to immune dysfunction, immunosuppression, infection, sepsis and MODS (4, 13, 19, 38–41). The disruption can lead to pathological interactions between monocyte, macrophage, NK and DCs, T cell dysfunction, and the development of persistent lymphopenia (8–10, 34, 38, 40, 42–45). Persistent lymphopenia carries a high mortality. Brohi’s group recently reported a 45% mortality rate in trauma patients when the lymphocyte count was ≤ 0.5 × 10^9^/L at 48 h after hospital admission (38). In addition, the type of trauma determines a patient’s susceptibility to persistent lymphopenia and infection, with traumatic brain injury (TBI) patients having disproportionally worse outcomes compared to those with burns, polytrauma or major surgery (43). A recent study of Campbell et al. reported that 37% of TBI patients were lymphopenic on hospital admission, and its persistence was associated with increased risk of mortality and pneumonia (46). Wang further reported that up to 83% of severe TBI patients contracted a respiratory infection within 3 days following injury (43, 47).

The mechanisms responsible for persistent lymphopenia and immunosuppression are not well understood (38, 48). The difficulty is that immunosuppression is a highly heterogeneous response involving differential T cell loss, T-cell exhaustion, T-helper 1 (Th1) depression, receptor shedding, and expansion of myeloid-derived suppressor cells (MDSCs) that have suppressive activity (44, 49). Separating the relative contributions of different immune cell subsets to post-traumatic immunosuppression has been a challenge. In a ground-breaking study, Mansen and colleagues examined early changes in circulating lymphocytes and showed that trauma patients who developed MODS within 24 h had nearly 2-fold higher CD56*^dim^* NK cells, 80% lower gamma delta (γδ)-low T cells and 4-fold higher IFN-γ upon hospital admission, compared to patients who did not (38). CD56*^dim^* NK cells are potent mediators of natural and antibody-dependent cytotoxicity and only weakly secrete cytokines (50). Moreover, the group showed that the patients who developed MODS also developed lymphopenia within 24 h of injury, which if persisted to 48 h led to high mortality (38). The association between lymphopenia, MODS and decreased frequencies and functional responses of innate T cells in trauma patients suggests that early immuno-inflammatory events may “predetermine” late secondary complications. The rise in NK cells and early fall in γδ-low T cells seen in patients who developed MODS may be clinically significant and predict risk for late complications, however, further studies are required (38, 48).

Another early driver of immune complications is HMGB1, which is a major DAMP that induces inflammation *via* TNF-α, IL-6, and IL-1β that in turn stimulate pattern recognition receptors TLR4 and RAGE on immune cells (Figure 2) (44, 51). In a rat polytrauma model (femoral osteotomy, blunt chest contusion and burn injury), Muire and colleagues showed that HMGB1 was an early contributor to the onset of lymphopenia and the loss of CD4^+^, CD8^+^, and γδ-T cells (34). Interestingly, the decrease in T cells was partly attenuated when HMGB1 was neutralized immediately post-trauma, however, the γδ-T cell population was not affected (51). The authors proposed that diminished levels of surface expression of RAGE and TLR4 on T cells, *via* ectodomain shedding, may be responsible for suppression *in vivo* (51). HMGB1 has also been shown to activate MDSCs after trauma and cancer (44), and is a late mediator of sepsis (44, 51), which further highlights the complexity of the system.

Apoptosis is believed to play a central role in persistent lymphopenia (52–58). Three main mechanisms for inducing lymphocyte apoptosis include: (1) cell-autonomous T-cell death (ACAD), (2) stress-related activation-induced cell death (AICD), and (3) newly discovered inflammasome-dependent monocyte activation (52–58) (Table 1). Persistently elevated plasma interleukin (IL)-10 levels have further been correlated with monocyte deactivation, reduced T cell activation and secondary infectious complications (8, 39, 40, 42, 59). Platelets also modulate T cell subsets *via* PAR4 that may link the innate and adaptive systems *via* pro-inflammatory cytokines (58). The interconnectedness of the T cell subsets and potential drivers of immunosuppression requires further research. Interestingly, post-injury immunosuppression shares many similarities with non-traumatic, sepsis-induced immunosuppression (41, 57).

**TABLE 1:** Possible mechanisms for T-cell apoptosis and immunosuppression after major trauma.

Pathway	Mechanisms	Comment	References
Cell-autonomous T cell death (ACAD)	• Intrinsic “caspase” pathway • Independent of death signals • Regulated by declining Bcl-2 at level of mitochondria • Cytochrome C released • Activates caspases • T cells undergo apoptosis without TCR restimulation	Toward the end of the immune response, activated lymphocytes not restimulated can die by permeabilizing the mitochondrial membrane. Bcl-2 is an anti-apoptotic protein that blocks the release of cytochrome c from mitochondria.	(53–55)
Stress-induced activation-induced cell death (AICD)	• Extrinsic “caspase” pathway • Death receptors: TNFR1, Fas, DR3, DR6, Trail-R1. • Glucocorticoid receptors (GRs) may also be involved • Receptor-driven apoptosis • Bax, bak, and BH3 domain • Activate caspases 8 and 3	A death receptor-mediated apoptosis pathway. The ligands for death receptors form a family of related cytokines collectively named as the TNF family.	(42, 52, 53, 56)
Monocyte-T cell interaction	• Extrinsic pathway • Inflammasome activation in monocytes sense DAMPs • IL-1β induced Fas-mediated monocyte driven T cell death *via* apoptosis	Monocytes sense injury-released DNA (DAMPs) *via* the AIM2 inflammasome and induce the extrinsic cell death of T cells.	(57)

## Central nervous system and organ interconnectedness: A major overlooked factor

The defense of the organism against deleterious agencies, which is at first confined to the phagocytic mechanisms and the somatic system of nerves, by and by spreads to and is undertaken by the psychical nervous apparatus … One function of these psychical cells has been to develop a complete science for the defense of the organism against hostile influences.

Metchnikoff (21) p. 195.

Metchnikoff had it right over 130 years ago. Activation of the “psychical cells” of the CNS following severe trauma results are important, and involve the release of norepinephrine, epinephrine and hormones (ACTH and glucocorticoids) from the adrenal medulla into the circulation and from the postganglionic nerve endings innervating the heart, and other organs of the body (14, 60–65). Traditionally, this is known as the whole-body stress response which dates back to Cannon (20, 66). The link between CNS injury, the immune system and immunosuppression is less well known. Yang and colleagues recently showed in a rat model of TBI that activation of sympathetic nervous system upregulated the expression of programmed cell death-1 (PD-1) on CD4^+^ and CD8^+^ T cells, and subsequently contributed to immunosuppression (43). The group speculated that immunosuppression may be partly mediated by stress hormones targeting β-adrenergic receptors (β-AR) on T cells (and indirectly B cells), because propranolol, a β-AR blocker, restored dysfunction *in vitro*, although they acknowledge it was more complex in the intact animal (43).

CNS modulation of the immune system occurs *via* the central hypothalamic-pituitary-adrenal (HPA) axis and the brainstem’s nucleus tractus solitarius (NTS) (67–70). After major trauma, the CNS balance switches to a sympathetic dominance and suppression of the parasympathetic system that normally counters inflammation *via* activation of the parasympathetic vagal cholinergic neurons and splanchnic/splenic nerves, known as the inflammatory reflex (71–73). The shift in CNS balance also impacts other organs, such as the heart and vasculature, and the gut microbiome *via* the gut-brain axis, which can alter blood flow to the gut wall and cause ischemia and increased permeability, where bacteria and/or their active metabolic products (lipopolysaccharides, cytokines, neuropeptides, and protein messengers) can enter the blood stream or lymph vessels and increase PAMPs and a patient’s susceptibility to infection (74, 75). Together, all these factors *may contribute to predetermining the extent and resolvability of the immuno-inflammatory response* after major trauma.

Another unappreciated fact in the polytrauma patient is that many undergo a second trauma from the corrective surgery itself (20). At all times, from the prehospital setting to after major surgery, the brain remains “wide awake” to changes in circulating DAMPs and PAMPs, inflammatory cytokines and immune cells circulating in the body (20, 76). Even the anesthetized brain remains “awake” because the blood brain barrier (BBB) is disrupted from the trauma and changes in cerebral blood flow and shear stress, which is part of the injury phenotype (77), and this is further amplified in the patient with a TBI (20, 74, 78, 79). Following any major trauma, the brain loses its “immune privilege” as it is no longer “separated” from the rest of the body (77). This is a research area in its infancy. We recently showed in a rat model of a laparotomy, designed to simulate a penetrating wound, that profound changes in gene expression occurred in brain, heart and other organs (80). Abdominal trauma was associated with 10–20-fold increases in plasma corticosterone, pro-inflammatory cytokines, endothelial injury markers, neutrophils (6 h), lactate (3 days), and coagulopathy (80). Lymphocytes decreased by ∼70% at 6 h and 3 days, and IL-10 dramatically increased from undetectable baseline levels to 483 pg/ml after 6 h and again at 3 days (1,149 pg/ml). Cortical excitability was high over 3 days with 30-fold increases in M1 muscarinic receptor expression and α-1A-adrenergic expression, and similar in heart with 8-fold increases in β-1-adrenergic receptor expression, and up to 6-fold increases in M2 and M1 muscarinic receptors after 6 h despite no changes in hemodymanics (80). These “silent” changes are remarkable given that there was only one incision, with no further injury to brain or heart. Unfortunately, we did not examine changes in the different T-cell subsets to further understand changes in immune activation.

## Systems hypothesis of trauma

Except on few occasions, the patient appears to die from the body’s response to infection rather than from it.

William Osler (81)

**FIGURE 3:**
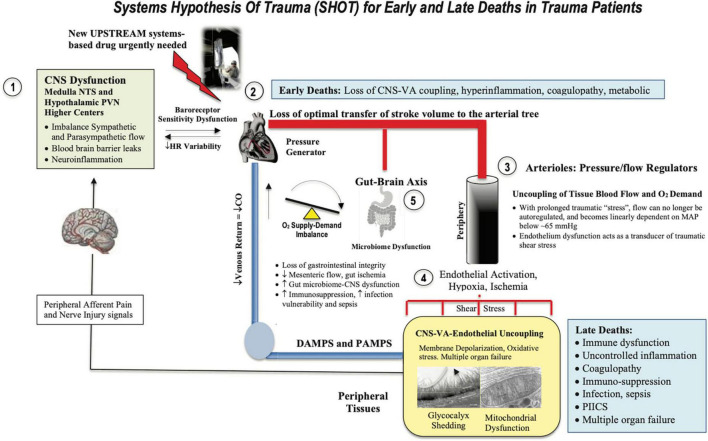
Systems Hypothesis Of Trauma (SHOT) showing key inter-connected sites of uncoupling during early and late deaths: (1) brain, (2) heart, (3) vasculature, (4) endothelial-glycocalyx-mitochondrial unit, and (5) gut barrier breaches. Our hypothesis is that if central and local control of cardiac output and ventriculo-arterial (VA) coupling are improved, endothelial and microvascular function will be improved and tissue O_2_ delivery will be maintained. An uncoupling is reflected by an increase in stress hormones, sympathetic discharge, loss of baroreceptor sensitivity, reduced heart rate variability (183, 184), unresolved inflammation, immune dysfunction, coagulopathy and mitochondrial dysfunction. New drugs are required to prevent inflammatory hyperdrive and support a VA-coupled, high flow, vasodilatory system with endothelial-glycocalyx protection and tissue oxygenation (185). HR, heart rate; ATP, adenosine triphosphate; CNS, central nervous system; NTS, nucleus tractus solitarius; PVN, paraventricular nucleus of the hypothalamus; MAP, mean arterial pressure. PIICS, Persistent Inflammation, Immunosuppression and Catabolism Syndrome, see text for description of early and late deaths.

Osler’s point cannot be overemphasized. It is not the infection that kills you it’s the body’s response to the trauma. The shift in homeostatic balance toward extreme limits and death led us to develop the Systems Hypothesis of Trauma (SHOT) (82) (Figure 3), which has undergone a number of iterations to include hemorrhagic trauma and the trauma of surgery (12, 13, 16, 20). SHOT has three pillars of protection.

1. CNS-cardiovascular coupling (Central Controller)
2. Endothelial glycocalyx material exchange (Systemic Integrator)
3. Mitochondrial integrity (Systemic Regulator)

## First pillar: Central nervous system-cardiovascular coupling

If the CNS can be protected early after trauma and the “hyperdrive” response can be suppressed, we argue that the immuno-inflammatory storms and lymphopenia will be reduced (20). According to SHOT, shifting autonomic balance toward parasympathetic outflows in the first minutes to hours after trauma would assist to maintain ventriculo-arterial (VA) coupling close to unity. VA coupling is a metric rarely discussed or measured in major trauma patients. It is the ratio of arterial elastance (Ea) to left-ventricular (LV) elastance (Ees) and can be measured from routine echocardiography (83–88). When the ratio is close to unity, the efficiency of the system is considered optimal. If the ratio is excessively high or low, the heart as a pump and vascular load become uncoupled with adverse downstream clinical outcomes (86, 89, 90). *The clinical advantage of VA coupling over ejection fraction (EF) or cardiac output (CO) is that it provides arterial load properties in addition to LV function* (86, 87). If the proximal arterial vessels become stiff, as a result of the CNS stress response, it increases load on the pump (91), whereas if the heart becomes stiff it cannot relax optimally to fill and eject blood into the conduit vessels (87). If both occur, they lead to VA uncoupling, tissue hypoperfusion, mitochondrial damage (92, 93) and subsequent immuno-inflammatory dysfunction (Figure 3). In the case of a high VA coupling ratio, vasodilator therapies can lower Ea and reduce the Ea/Ees ratio toward 1.0, and in the case of a low ratio, inotropes can increases Ees to improve VA coupling (92).

We predict further that maintaining VA coupling would improve immune function by reducing gut-brain axis dysfunction and preventing the gut wall from becoming ischemic and leaky which exacerbates immuno-inflammatory conditions, coagulopathy, immunosuppression, infection and sepsis (75). Howard and colleagues reported in trauma patients rapid changes in the microbiome during resuscitation and stabilization (94), although further studies are required to understand the role of the gut in exacerbating systemic inflammation and infectious complications after major trauma.

## Second pillar: The endothelial glycocalyx

The second pillar of SHOT is to maintain the health of the endothelium (95). The endothelium is located at the nexus of the blood and tissues and controls the transfer of O_2_, metabolic fuels, hormones, immune cells and factors, inflammatory regulators and fluids (96–102). Trauma-induced damage to this organ is termed the endotheliopathy of trauma (EoT), which is characterized by endothelial activation, vasoactivity, loss of barrier function, leukocyte adhesion, coagulopathy, inflammation and organ dysfunction (103–110). In addition, the endothelium, like the BBB, is highly sensitive to changes in blood flow and shear stress, which can alter vascular tone, tissue perfusion, exchange and permeability (111).

**FIGURE 4:**
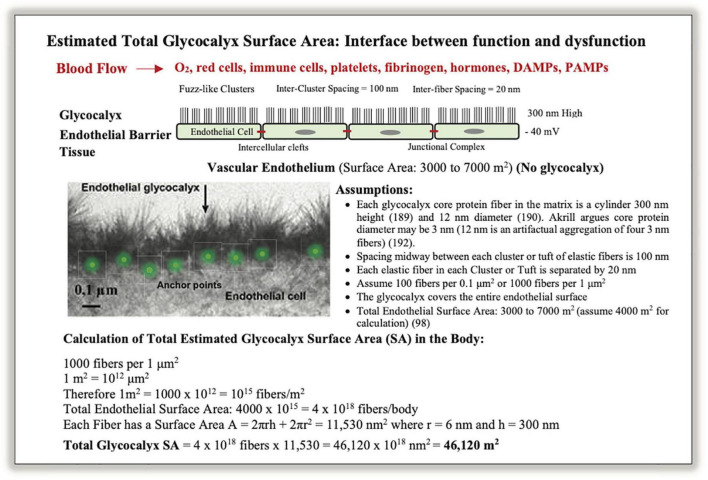
A schematic of the vascular endothelium and calculation of total glycocalyx surface area (SA) in humans. Photo insert was modified from Chappell et al. (186). Glycocalyx fibers appear in clusters composed of proteoglycans, glycosaminoglycans, and glycoproteins, which are anchored into endothelial cells by core-proteins (113, 115, 187, 188). Together they form a dynamic structure that participates in shear stress regulation, barrier protection, vascular permeability, inflammation, coagulopathy, fibrinolysis, mechanotransduction, immune function and cytokine signaling (97, 101, 104, 116). The glycocalyx is difficult to characterize because of its fragility and instability, and its structural dimensions critically depend on the method of ultrastructural visualization (97, 116, 189–193). The SA calculation should be viewed as approximate. A glycocalyx SA of 44,120 m^2^ for material exchange equates to ∼200 tennis courts or over 8 US football fields (see text).

The endothelial surface area (SA) has been estimated to be 3,000–7,000 m^2^ (98, 112). However, this estimate ignores the SA of the glycocalyx mesh that is synthesized and secreted by the endothelium and anchored to its cellular lining. As mentioned, the function of the endothelial glycocalyx is dynamic and diverse and it also acts as a vascular filter overlying the endothelial cell-cell junctions as it contains a large volume of non-circulating plasma (1–1.7 L) (113–115). We have estimated for the first time the SA of glycocalyx and found it was at least 10-fold higher than the endothelium (SA_*glycocalyx*_ = 46,120 m^2^) (see Figure 4). This is equivalent to a SA of over ∼200 tennis courts or 8 USA football fields, and given its central role has major implications to immune function and secondary injury progression post-trauma.

When damaged by inflammatory mechanisms, the endothelium can rapidly shed its glycocalyx “fuzz” *via* sheddases, and release nanoscale bioactives and DAMPs, such as thrombomodulin, syndecan-1, heparan sulfate, hyaluronic acid, and other proteoglycans and glycoproteins, into the circulation (96, 104, 107, 116–119). This degradation is believed to perpetuate immuno-inflammation and coagulopathy (13, 120–125), immunosuppression (102, 126, 127) and mitochondrial dysfunction (115, 128, 129). SHOT predicts if VA coupling is close to unity and tissue perfusion and O_2_ exchange can be maintained, damage to the endothelium-glycocalyx will be minimized and these secondary injury processes reduced (Figure 3). Remarkably, if adequate tissue perfusion can be restored, the damaged glycocalyx has the capacity to repair itself (130, 131). Timing of repair appears to depend upon the duration and extent of hypoperfusion and ischemia, and the type and severity of trauma (130, 131).

## Third pillar: Mitochondrial integrity

Maintaining the functional integrity of mitochondria post-trauma is essential for a good outcome. Mitochondria are sensor organelles of ancient bacterial origins involved in ATP production, substrate regulation, immune cell signaling, calcium homeostasis, endoplasmic reticulum communication, and cell death regulation (32, 132–135). After severe trauma, prolonged hypoperfusion leads to mitochondrial damage. However, before damage occurs there is a switch from aerobic mitochondrial oxidative and to anaerobic glycolytic metabolism, which can only be sustained for short periods of time (136). Damage occurs from depletion of local glycogen stores, depolarization of the sarcolemma membrane, increased lactate, reduced pH, increases in cell Ca^2+^ loading, a fall in ATP phosphorylation and redox potentials, increased reactive oxygen species, reduced inner mitochondrial membrane proton pumping, opening of the permeability transition pore, collapse of mitochondrial membrane potential and finally the release of cytochrome C, and other DAMPs (32, 134, 135, 137–142). DAMPs from damaged mitochondria exacerbate CNS injury, cardiovascular dysfunction and secondary injury (143–145). According to SHOT, improving CNS protection and CNS-cardiovascular-endothelium coupling will improve tissue perfusion and protect mitochondrial integrity (146) (Figure 3).

## Other unifying models of traumatic injury

In 2017, Johansson and colleagues introduced a model of SHock-INduced Endotheliopathy (SHINE) to better understand the underlying pathophysiological mechanisms for critically ill patients (147). Like SHOT, they propose that shock-induced sympatho-adrenal hyperactivation is a critical driver of endothelial cell and glycocalyx damage, hypoperfusion, and subsequent hemostatic aberrations and multiorgan dysfunction (110). More recently, Henriksen reported that patients with identical trauma severity developed *significantly different degrees of endothelial dysfunction*, as measured by syndecan-1, and proposed a minimum of four shock-induced endotheliopathy phenotypes (148) with the differences most likely driven by a genetic component (148). Moreover, they introduced a new research tool in trauma by using metabolic systems biology, which should be encouraged. A major difference between SHOT and SHINE is the functional linkage between CNS and VA coupling, which is testable. SHINE does not include this key linkage, which describes the coupling of cardiac and arterial vascular reactivity to optimally propel blood to deliver sufficient oxygen from the lungs to tissue mitochondria and prevent and/or reduce ongoing immuno-inflammatory dysfunction (discussed above).

## Urgent need for systems-based therapies: Heterogeneity vs. homogeneity in research

How do we switch the injury phenotype of a polytrauma patient to a survival one? Why are there no effective drugs to treat immune dysregulation in the early hours to days following major trauma or in the critically ill patient? We argue the main reasons for lack of progress in drug development include:

1. Failure to replicate the heterogeneity of humans.
2. Poorly designed trials lacking diversity.
3. Inappropriate use of pathogen-free animals.
4. Ignoring sex-specific differences.
5. The flawed practice of single-nodal targeting.

The heterogeneity of the human condition is a major variable when conducting animal experiments to solve a medical problem (149). Preclinical models typically use animals from relatively homogeneous breeding colonies whereas humans are genetically, epigenetically, biologically and physiologically heterogeneous (149, 150). Large animals, such as pigs and sheep, do have some advantages with similar physiologies and/or anatomies as humans, however, they are more costly than using rodents (149, 150). A second confounding variable are poorly designed human trials that are either not sufficiently powered or recruit patients who do not adequately represent the wider population for which the drug therapy is intended (151–153).

Similar problems apply to preclinical models that use specific pathogen-free (SPF) animals. SPF animals were introduced in the early 1960s to minimize disease or infection as an unwanted variable in experimental design (151, 154). However, SPF animals have different gut microbiota that can profoundly influence basic physiology, stress behaviors and the immuno-inflammatory response to trauma (151–153, 155). Beura et al. showed that SPF adult mice, for example, have “immature” immune systems that were more prone to infection than conventionally bred mice (156). SPF animals may be useful for studying specific questions in biochemical mechanisms, but they do not mimic the patient following trauma (152). The current consensus is that conventionally bred animals are the animals of choice if translation of a new drug therapy is the end-game (151). In addition, the mouse model may be problematic for trauma studies because unlike rats, guinea-pigs, pigs, sheep, dogs, and humans, mice can enter a dormant state, called torpor, when subjected to traumatic stress (157, 158). Torpor itself can profoundly change the animal’s immune system by reducing the numbers of circulating leukocytes, lowering complement levels, and changing the animal’s response to infection (159).

The other important variable in preclinical and clinical studies is sex. An increasing number of animal and human studies show sex-specific differences in pathophysiological responses to polytrauma, hemorrhagic shock, TBI and burns (160–162). Chaudry and colleagues have been emphasizing the importance of sex in biochemical research for over two decades. They showed that administration of female sex hormone 17β-estradiol in males and ovariectomized females after trauma-hemorrhage prevented the suppression of immune response (163, 164). On the basis of accumulated data, greater inclusion of females in preclinical modeling and translation has been earmarked by the National Institutes of Health (NIH), European Commission, US Department of Defense and FDA (151–153).

Lastly, the practice of single-nodal targeting is another factor for why there are no effective systems-acting drugs for the polytrauma patient. Past drug development efforts have focused more on alleviating symptoms rather than addressing an underlying problem. The current practice of treat-as-you-go using sequential, single-target therapies leads to what US surgeon William C. Shoemaker considered: “an uncoordinated and sometimes contradictory therapeutic outcome” (165). Targeting individual pro-inflammatory cytokines, or any single step along a signaling pathway, ignores the critical importance of the system. Single-nodal thinking rarely solves a medical problem unless the site is believed to be a central hub or upstream intersection point. The IL-1 receptor has been proposed to be such a target, and while *anakinra* (IL-1 antagonist) has an excellent safety record, further trials are required to demonstrate its clinical efficacy after trauma or infection (166, 167). Reductionism in scientific discovery is important in breaking a system into its constituent parts, however, *it does not do away with the system* (151–153). This flawed way of thinking, we believe, is a major contributor for the high failure rate of translating promising new drugs in clinical trials (168). Choosing the right model and experimental design, a systems approach is much more likely to increase animal-to-human translational success to improve trauma care.

## Adenosine, lidocaine and magnesium (ALM): Toward a systems-based drug therapy

If you control hemorrhage and infection, the patient will do the recovery, since every cell in his body is working hard in that direction already. But you must understand what those cells are doing so that you can help them.

Walter B. Cannon [Moore, (169) p. 816].

We have been developing a small-volume intravenous (IV) ALM fluid therapy to treat polytrauma for civilian and military use (12, 16). In different animal models, ALM confers a survival advantage after hemorrhagic shock (12, 16, 170, 171), traumatic injury (170–174), sepsis (175, 176) and endotoxin insult (177). The ALM survival phenotype is not replicated with individual actives adenosine, lidocaine or magnesium (12, 16). ALM confers its benefit by shifting CNS function from sympathetic to parasympathetic dominance (178), blunting inflammation (172), correcting coagulopathy (179), maintaining VA coupling, improving tissue blood flow, lowering energy demand and protecting mitochondria (178). Studies carried out by US Army Institute of Surgical Research also showed that ALM therapy restored 97% of endothelial glycocalyx after severe hemorrhagic shock (180). Currently, we don’t know how and when the “switch” from an injury phenotype to a survival phenotype occurs, however, we suspect it is early because the same 5 h therapy confers dual protection against trauma and infection (12, 14, 16). It is possible ALM may act in the first minutes to hours after administration to assist the body to develop a “normal” immune response with timely resolution of the immuno-inflammatory genomic storms. While the preclinical ALM data appear promising, translation to humans remains challenging given the failure rate of translating new drugs into humans exceeds 95% (181), and of those that do obtain FDA approval, around 30% show postmarket safety concerns (182). Understanding the underlying mechanisms of action of ALM is vital for safe translation.

## Conclusion

Trauma is a leading cause of death and disability worldwide. Currently there are no effective drug therapies to reduce hyperinflammation and immune dysfunction, immunosuppression, infection and MODS following major trauma. The present treat-as-you-go approaches fail to appreciate that immuno-inflammatory complications are a systems failure, and not a single nodal failure. New therapies are required to target the CNS control of cardiovascular function, endothelial-glycocalyx shedding, tissue O_2_ supply and its mitochondrial circuitry in both homeostatic and pathophysiological processes to prevent those complications.

## Author contributions

GD: concept. GD, JM, and HL: data collection, data analyses, interpretation, and manuscript preparation and editing. All authors contributed equally to the design, implementation, literature analysis and writing of the manuscript.

## References

[1] Bedard AF Mata LV Dymond C Moreira F Dixon J Schauer SG A scoping review of worldwide studies evaluating the effects of prehospital time on trauma outcomes. *Int J Emerg Med.* (2020) 13:64. 10.1186/s12245-020-00324-7 33297951 PMC7724615

[2] Dobson GP Morris JL Letson HL. Why are bleeding trauma patients still dying? Towards a systems hypothesis of trauma. *Front Physiol.* (2022) 13:990903. 10.3389/fphys.2022.990903 PMC9485567 36148305

[3] Rauf R Von Matthey F Croenlein M Zyskowski M Van Griensven M Biberthaler P Changes in the temporal distribution of in-hospital mortality in severely injured patients-an analysis of the TraumaRegister DGU. *PLoS One.* (2019) 14:e0212095. 10.1371/journal.pone.0212095 30794579 PMC6386341

[4] Brohi K Gruen RL Holcomb JB. Why are bleeding trauma patients still dying? *Intensive Care Med.* (2019) 45:709–11. 10.1007/s00134-019-05560-x 30741331

[5] Lee C Rasmussen TE Pape HC Gary JL Stannard JP Haller JM. The polytrauma patient: Current concepts and evolving care. *OTA Int.* (2021). 4:e108–14. 10.1097/OI9.0000000000000108 PMC10441682 37608855

[6] Lansink KW Gunning AC Leenen LP. Cause of death and time of death distribution of trauma patients in a Level I trauma centre in the Netherlands. *Eur J Trauma Emerg Surg.* (2013) 39:375–83. 10.1007/s00068-013-0278-2 26815398

[7] Rosenthal MD Moore FA. Persistent Inflammation, Immunosuppression, and catabolism: evolution of multiple organ dysfunction. *Surg Infect (Larchmt).* (2016) 17:167–72. 10.1089/sur.2015.184 26689501 PMC4790202

[8] Efron PA Mohr AM Bihorac A Horiguchi H Hollen MK Segal MS Persistent inflammation, immunosuppression, and catabolism and the development of chronic critical illness after surgery. *Surgery.* (2018) 164:178–84. 10.1016/j.surg.2018.04.011 29807651 PMC6056337

[9] Osuka A Ogura H Ueyama M Shimazu T Lederer JA. Immune response to traumatic injury: harmony and discordance of immune system homeostasis. *Acute Med Surg.* (2014) 1:63–9. 10.1002/ams2.17 29930824 PMC5997205

[10] Mas-Celis F Olea-Lopez J Parroquin-Maldonado JA. Sepsis in trauma: a deadly complication. *Arch Med Res.* (2021) 52:808–16. 10.1016/j.arcmed.2021.10.007 34706851

[11] Kotwal RS Staudt AM Trevino JD Valdez-Delgado KK Le TD Gurney JM A review of casualties transported to role 2 medical treatment facilities in Afghanistan. *Mil Med.* (2018) 183(Suppl. 1):134–45. 10.1097/01.ccm.0000528322.39346.3a 29635602

[12] Dobson GP Letson HL. Far forward gaps in hemorrhagic shock and prolonged field care: an update of ALM fluid therapy for field use. *J Spec Oper Med.* (2020) 20:78–84. 10.55460/06VT-9IH4 32969018

[13] Dobson GP Morris JL Davenport LM Letson HL. Traumatic-induced coagulopathy as a systems failure: a new window into hemostasis. *Semin Thromb Hemost.* (2020) 46:199–214. 10.1055/s-0039-1701018 32069514

[14] Dobson GP Biros E Letson HL Morris JL. Living in a hostile world: inflammation, new drug development and coronavirus. *Front Immunol.* (2021) 11:610131. 10.3389/fimmu.2020.610131 33552070 PMC7862725

[15] Cannon WB. Organisation for physiological homeostasis. *Physiol Rev.* (1929) 9:399–431. 10.1152/physrev.1929.9.3.399

[16] Dobson GP Letson HL. Adenosine, lidocaine and Mg2+ (ALM): from cardiac surgery to combat casualty care: teaching old drugs new tricks. *J Trauma Acute Care Surg.* (2016) 80:135–45. 10.1097/TA.0000000000000881 26683400

[17] Dobson GP. Organ arrest, protection and preservation: natural hibernation to cardiac surgery: a review. *Comp Biochem Physiol Part B.* (2004) 139:469–85. 10.1016/j.cbpc.2004.06.002 15544969

[18] Egiazaryan GG Sudakov KV. Theory of functional systems in the scientific school of P.K. Anokhin. *J Hist Neurosci.* (2007) 16:194–205. 10.1080/09647040600602805 17365564

[19] Carlson DL Horton JW. Cardiac molecular signaling after burn trauma. *J Burn Care Res.* (2006) 27:669–75. 10.1097/01.BCR.0000237955.28090.41 16998399

[20] Dobson GP. Trauma of major surgery: a global problem that is not going away. *Int J Surg.* (2020) 81:47–54. 10.1016/j.ijsu.2020.07.017 32738546 PMC7388795

[21] Metchnikoff E. Lectures on the comparative pathology of inflammation, delivered at the Pasteur institute in 1891. In: Metchnikoff E editor. *Lectures on the Comparative Pathology of Inflammation. (Translated by F. A. Starling and E. H. Starling.).* New York, NY: Dover Publications (1968).

[22] Chou C Li MO. Tissue-resident lymphocytes across innate and adaptive lineages. *Front Immunol.* (2018) 9:2104. 10.3389/fimmu.2018.02104 30298068 PMC6160555

[23] Fan X Rudensky AY. Hallmarks of tissue-resident lymphocytes. *Cell* (2016) 164:1198–211. 10.1016/j.cell.2016.02.048 26967286 PMC4973889

[24] Roehr B. Tissue resident memory cells emerging as key player in health and disease. *Proc Natl Acad Sci USA.* (2017) 114:12092–3. 10.1073/pnas.1715754114 29138347 PMC5699094

[25] Merle NS Church SE Fremeaux-Bacchi V Roumenina LT. Complement system part I - molecular mechanisms of activation and regulation. *Front Immunol.* (2015) 6:262. 10.3389/fimmu.2015.00262 26082779 PMC4451739

[26] Klose CSN Artis D. Innate lymphoid cells control signaling circuits to regulate tissue-specific immunity. *Cell Res.* (2020) 30:475–91. 10.1038/s41422-020-0323-8 32376911 PMC7264134

[27] Galli SJ Gaudenzio N. Human mast cells as antigen-presenting cells: when is this role important in vivo? *J Allergy Clin Immunol.* (2018) 141:92–3. 10.1016/j.jaci.2017.05.029 28624609

[28] Dorrington MG Fraser IDC. NF-kappaB signaling in macrophages: dynamics, crosstalk, and signal integration. *Front Immunol.* (2019) 10:705. 10.3389/fimmu.2019.00705 31024544 PMC6465568

[29] Allie SR Randall TD. Resident memory B cells. *Viral Immunol.* (2020). [Epub ahead of print]. 10.1089/vim.2019.0141 32023188 PMC7247033

[30] Bianchi ME. DAMPs, PAMPs and alarmins: all we need to know about danger. *J Leukoc Biol.* (2007) 81:1–5. 10.1189/jlb.0306164 17032697

[31] Venereau E Ceriotti C Bianchi ME. DAMPs from cell death to new life. *Front Immunol.* (2015) 6:422. 10.3389/fimmu.2015.00422 26347745 PMC4539554

[32] Hauser CJ Otterbein LE. Danger signals from mitochondrial DAMPS in trauma and post-injury sepsis. *Eur J Trauma Emerg Surg.* (2018) 44:317–24. 10.1007/s00068-018-0963-2 29797026

[33] Roh JS Sohn DH. Damage-associated molecular patterns in inflammatory diseases. *Immune Netw.* (2018) 18:e27. 10.4110/in.2018.18.e27 30181915 PMC6117512

[34] Muire PJ Schwacha MG Wenke JC. Systemic T cell exhaustion dynamics is linked to early high mobility group box protein 1 (HMGB1) driven hyper-inflammation in a polytrauma rat model. *Cells.* (2021) 10:1646. 10.3390/cells10071646 34209240 PMC8305113

[35] Piccinini AM Midwood KS. DAMPening inflammation by modulating TLR signalling. *Mediators Inflamm.* (2010) 2010:1–21. 10.1155/2010/672395 20706656 PMC2913853

[36] Xiao W Mindrinos MN Seok J Cuschieri J Cuenca AG Gao H A genomic storm in critically injured humans. *J Exp Med.* (2011) 208:2581–90. 10.1084/jem.20111354 22110166 PMC3244029

[37] Gentile LF Cuenca AG Efron PA Ang D Bihorac A Mckinley BA Persistent inflammation and immunosuppression: a common syndrome and new horizon for surgical intensive care. *J Trauma Acute Care Surg.* (2012) 72:1491–501. 10.1097/TA.0b013e318256e000 22695412 PMC3705923

[38] Manson J Cole E De’ath HD Vulliamy P Meier U Pennington D Early changes within the lymphocyte population are associated with the development of multiple organ dysfunction syndrome in trauma patients. *Crit Care.* (2016) 20:176. 10.1186/s13054-016-1341-2 27268230 PMC4895987

[39] Thompson KB Krispinsky LT Stark RJ. Late immune consequences of combat trauma: a review of trauma-related immune dysfunction and potential therapies. *Mil Med Res.* (2019) 6:11. 10.1186/s40779-019-0202-0 31014397 PMC6480837

[40] Sturm R Xanthopoulos L Heftrig D Oppermann E Vrdoljak T Dunay IR Regulatory T cells modulate CD4 proliferation after severe trauma via IL-10. *J Clin Med.* (2020) 9:1052. 10.3390/jcm9041052 32276346 PMC7230720

[41] Vazquez AC Arriaga-Pizano L Ferat-Osorio E. Cellular markers of immunosuppression in sepsis. *Arch Med Res.* (2021) 52:828–35. 10.1016/j.arcmed.2021.10.001 34702587

[42] Kimura F Shimizu H Yoshidome H Ohtsuka M Miyazaki M. Immunosuppression following surgical and traumatic injury. *Surg Today.* (2010) 40:793–808. 10.1007/s00595-010-4323-z 20740341 PMC7101797

[43] Yang Y Ye Y Chen C Kong C Su X Zhang X Acute traumatic brain injury induces CD4+ and CD8+ T cell functional impairment by upregulating the expression of PD-1 via the activated sympathetic nervous system. *Neuroimmunomodulation.* (2019) 26:43–57. 10.1159/000495465 30695785

[44] Ruan X Darwiche SS Cai C Scott MJ Pape HC Billiar TR. Anti-HMGB1 monoclonal antibody ameliorates immunosuppression after peripheral tissue trauma: attenuated T-lymphocyte response and increased splenic CD11b (+) Gr-1 (+) myeloid-derived suppressor cells require HMGB1. *Mediators Inflamm.* (2015) 2015:458626. 10.1155/2015/458626 25709155 PMC4325468

[45] Islam MN Bradley BA Ceredig R. Sterile post-traumatic immunosuppression. *Clin Transl Immunol.* (2016) 5:e77. 10.1038/cti.2016.13 27195120 PMC4855263

[46] Campbell B Budreau D Williams-Perez S Chakravarty S Galet C Mcgonagill P. Admission lymphopenia predicts infectious complications and mortality in traumatic brain injury victims. *Shock.* (2022) 57:189–98. 10.1097/SHK.0000000000001872 34618726

[47] Wang J-W Li J-P Song Y-L Zhao Q-H. Humoral and cellular immunity changed after traumatic brain injury in human patients. *Ann Clin Lab Sci.* (2017) 47:10–6. 28249910

[48] Bonneville M O’Brien RL Born WK. Gammadelta T cell effector functions: a blend of innate programming and acquired plasticity. *Nat Rev Immunol.* (2010) 10:467–78. 10.1038/nri2781 20539306

[49] Gabrilovich DI Nagaraj S. Myeloid-derived suppressor cells as regulators of the immune system. *Nat Rev Immunol.* (2009) 9:162–74. 10.1038/nri2506 19197294 PMC2828349

[50] Muller-Heck RM Bosken B Michiels I Dudda M Jager M Flohe SB. Major surgical trauma impairs the function of natural killer cells but does not affect monocyte cytokine synthesis. *Life (Basel).* (2021) 12:13. 10.3390/life12010013 35054405 PMC8777869

[51] Yang H Wang H Andersson U. Targeting inflammation driven by HMGB1. *Front Immunol.* (2020) 11:484. 10.3389/fimmu.2020.00484 32265930 PMC7099994

[52] Kumar R Herbert PE Warrens AN. An introduction to death receptors in apoptosis. *Int J Surg.* (2005) 3:268–77. 10.1016/j.ijsu.2005.05.002 17462297

[53] Arnold R Brenner D Becker M Frey CR Krammer PH. How T lymphocytes switch between life and death. *Eur J Immunol.* (2006) 36:1654–8. 10.1002/eji.200636197 16791883

[54] Krammer PH Arnold R Lavrik IN. Life and death in peripheral T cells. *Nat Rev Immunol.* (2007) 7:532–42. 10.1038/nri2115 17589543

[55] Brenner D Krammer PH Arnold R. Concepts of activated T cell death. *Crit Rev Oncol Hematol.* (2008) 66:52–64. 10.1016/j.critrevonc.2008.01.002 18289867

[56] Fulda S Gorman AM Hori O Samali A. Cellular stress responses: cell survival and cell death. *Int J Cell Biol.* (2010) 2010:214074. 10.1155/2010/214074 20182529 PMC2825543

[57] Roth S Cao J Singh V Tiedt S Hundeshagen G Li T Post-injury immunosuppression and secondary infections are caused by an AIM2 inflammasome-driven signaling cascade. *Immunity.* (2021) 54:648–659.e8. 10.1016/j.immuni.2021.02.004 33667383

[58] Bock M Bergmann CB Jung S Biberthaler P Heimann L Hanschen M. Platelets differentially modulate CD4(+) Treg activation via GPIIa/IIIb-, fibrinogen-, and PAR4-dependent pathways. *Immunol Res.* (2022) 70:185–96. 10.1007/s12026-021-09258-5 34932195 PMC8917040

[59] Abe R Hirasawa H Oda S Sadahiro T Nakamura M Watanabe E Up-regulation of interleukin-10 mRNA expression in peripheral leukocytes predicts poor outcome and diminished human leukocyte antigen-DR expression on monocytes in septic patients. *J Surg Res.* (2008) 147:1–8. 10.1016/j.jss.2007.07.009 17720196

[60] Desborough JP. The stress response to trauma and surgery. *Br J Anaesth.* (2000) 85:109–17. 10.1093/bja/85.1.109 10927999

[61] Ballard-Croft C Maass DL Sikes P White JM Horton J. Activation of stress-responsive pathways by the sympathetic nervous system in burn trauma. *Shock.* (2002) 18:38–45. 10.1097/00024382-200207000-00008 12095132

[62] Sternberg EM. Neural regulation of innate immunity: a coordinated nonspecific host response to pathogens. *Nat Rev Immunol.* (2006) 6:318–28. 10.1038/nri1810 16557263 PMC1783839

[63] Hotamisligil GS Davis RJ. Cell signaling and stress responses. *Cold Spring Harb Perspect Biol.* (2016) 8:a006072. 10.1101/cshperspect.a006072 27698029 PMC5046695

[64] Powell K Shah K Hao C Wu YC John A Narayan RK Neuromodulation as a new avenue for resuscitation in hemorrhagic shock. *Bioelectron Med.* (2019) 5:17. 10.1186/s42234-019-0033-z 32232106 PMC7098257

[65] Gans IM Coffman JA. Glucocorticoid-mediated developmental programming of vertebrate stress responsivity. *Front Physiol.* (2021) 12:812195. 10.3389/fphys.2021.812195 34992551 PMC8724051

[66] Prete A Yan Q Al-Tarrah K Akturk HK Prokop LJ Alahdab F The cortisol stress response induced by surgery: a systematic review and meta-analysis. *Clin Endocrinol (Oxf).* (2018) 89:554–67. 10.1111/cen.13820 30047158

[67] Smith SM Vale WW. The role of the hypothalamic-pituitary-adrenal axis in neuroendocrine responses to stress. *Dialog Clin Neurosci.* (2006) 8:383–95. 10.31887/DCNS.2006.8.4/ssmith PMC3181830 17290797

[68] Charkoudian N Wallin BG. Sympathetic neural activity to the cardiovascular system: integrator of systemic physiology and interindividual characteristics. *Compr Physiol.* (2014) 4:825–50. 10.1002/cphy.c130038 24715570

[69] Burford NG Webster NA Cruz-Topete D. Hypothalamic-pituitary-adrenal axis modulation of glucocorticoids in the cardiovascular system. *Int J Mol Sci.* (2017) 18:2150. 10.3390/ijms18102150 29035323 PMC5666832

[70] Barman SM. 2019 Ludwig lecture: rhythms in sympathetic nerve activity are a key to understanding neural control of the cardiovascular system. *Am J Physiol Regul Integr Comp Physiol.* (2020) 318:R191–205. 10.1152/ajpregu.00298.2019 31664868 PMC7052600

[71] Pavlov VA Tracey KJ. Neural regulators of innate immune responses and inflammation. *Cell Mol Life Sci.* (2004) 61:2322–31. 10.1007/s00018-004-4102-3 15378203 PMC11138906

[72] Huston JM. The vagus nerve and the inflammatory reflex: wandering on a new treatment paradigm for systemic inflammation and sepsis. *Surg Infect (Larchmt).* (2012) 13:187–93. 10.1089/sur.2012.126 22913335

[73] Tracey KJ. The inflammatory reflex. *Nature.* (2020) 420:853–9. 10.1038/nature01321 12490958

[74] Sudo N. Microbiome, HPA axis and production of endocrine hormones in the gut. In: Lyte M Cryan J editors. *Microbiology Endocrinology: The Microbiota-Gut Brain Axis in Health and Disease.* New York, NY: Springer (2014). 10.1007/978-1-4939-0897-4_8

[75] Mayer EA Tillisch K Gupta A. Gut/brain axis and the microbiota. *J Clin Invest.* (2015) 125:926–38. 10.1172/JCI76304 25689247 PMC4362231

[76] Crile G. Nitrous oxide anaesthesia and a note on anoci-association, a new principle in operative surgery. *Surg Gynecol Obstet.* (1911) 13:170–3.

[77] Montagne A Toga AW Zlokovic BV. Blood-brain barrier permeability and gadolinium: benefits and potential pitfalls in research. *JAMA Neurol.* (2016) 73:13–4. 10.1001/jamaneurol.2015.2960 26524294 PMC4736734

[78] Turnbull AV Rivier C. Regulation of the HPA axis by cytokines. *Brain Behav Immun.* (1995) 9:253–75. 10.1006/brbi.1995.1026 8903845

[79] Kadry H Noorani B Cucullo L. A blood-brain barrier overview on structure, function, impairment, and biomarkers of integrity. *Fluids Barriers CNS.* (2020) 17:69. 10.1186/s12987-020-00230-3 33208141 PMC7672931

[80] Dobson GP Morris JL Biros E Davenport LM Letson HL. Major surgery leads to a proinflammatory phenotype: differential gene expression following a laparotomy. *Ann Med Surg (Lond).* (2021) 71:102970. 10.1016/j.amsu.2021.102970 34745602 PMC8554464

[81] Osler W. *The Evolution of Modern Medicine*. New Haven, CT: Yale, University Press (1921). p. 243.

[82] Dobson GP. Addressing the global burden of trauma in major surgery. *Front Surg.* (2015) 2:43. 10.3389/fsurg.2015.00043 26389122 PMC4558465

[83] Suga H Goto Y Kawaguchi O Hata K Takasago T Saeki TW Ventricular perspective on efficiency. In: Burkhoff D Schaefer J Schaffner K Yue DT editors. *Myocardial Optimization and Efficiency, Evolutionary Aspects and Philosophy of Science Considerations.* New York, NY: Springer-Verlag (1993). p. 43–65.

[84] London GM. Role of arterial wall properties in the pathogenesis of systolic hypertension. *Am J Hypertens.* (2005) 18(1 Pt 2):19S–22S. 10.1016/j.amjhyper.2004.10.001 15683728

[85] Kass DA. Ventricular arterial stiffening: integrating the pathophysiology. *Hypertension.* (2005) 46:185–93. 10.1161/01.HYP.0000168053.34306.d4 15911741

[86] Guarracino F Baldassarri R Pinsky MR. Ventriculo-arterial decoupling in acutely altered hemodynamic states. *Crit Care.* (2013) 17:213–20. 10.1186/cc12522 23510336 PMC3672525

[87] Cholley B Le Gall A. Ventriculo-arterial coupling: the comeback? *J Thorac Dis.* (2016) 8:2287–9. 10.21037/jtd.2016.08.34 27746956 PMC5059291

[88] Dobson GP Arsyad A Letson HL. The adenosine hypothesis revisited: a possible role for arterial compliance and its implications to coronary perfusion. *Front Physiol.* (2017) 8:824. 10.3389/fphys.2017.00824 29104545 PMC5654924

[89] Onorati F Santini F Dandale R Ucci G Pechlivanidis K Menon T “Polarizing” microplegia improves cardiac cycle efficiency after CABG for unstable angina. *Int J Cardiol.* (2013) 167:2739–46. 10.1016/j.ijcard.2012.06.099 22795715

[90] Granfeldt A Letson HL Hyldebrandt JA Wang ER Salcedo PA Nielson TK Small-volume 7.5% NaCl adenosine, lidocaine and Mg2+ has multiple benefits during hypotensive and blood resuscitation in the pig following severe blood loss: rat to pig translation. *Crit Care Med.* (2014) 42:e329–44. 10.1097/CCM.0000000000000225 24557427

[91] Ye Z Coutinho T Pellikka PA Villarraga HR Borlaug BA Kullo IJ. Associations of alterations in pulsatile arterial load with left ventricular longitudinal strain. *Am J Hypertens.* (2015) 28:1325–31. 10.1093/ajh/hpv039 25840581 PMC4715245

[92] Antonini-Canterin F Poli S Vriz O Pavan D Bello VD Nicolosi GL. The ventricular-arterial coupling: from basic pathophysiology to clinical application in the echocardiography laboratory. *J Cardiovasc Echogr.* (2013) 23:91–5. 10.4103/2211-4122.127408 28465893 PMC5353400

[93] Ky B French B May Khan A Plappert T Wang A Chirinos JA Ventricular-arterial coupling, remodeling, and prognosis in chronic heart failure. *J Am Coll Cardiol.* (2013) 62:1165–72. 10.1016/j.jacc.2013.03.085 23770174 PMC3943424

[94] Howard BM Kornblith LZ Christie SA Conroy AS Nelson MF Campion EM Characterizing the gut microbiome in trauma: significant changes in microbial diversity occur early after severe injury. *Trauma Surg Acute Care Open.* (2017) 2:e000108. 10.1136/tsaco-2017-000108 29766103 PMC5877916

[95] Wiel E Vallet B ten Cate H. The endothelium in intensive care. *Crit Care Clin.* (2005) 21:403–16. 10.1016/j.ccc.2005.03.001 15992664

[96] Bennett HS. Morphological aspects of extracellular polysaccharides. *J Histochem Cytochem.* (1963) 11:14–23. 10.1177/11.1.14

[97] Luft JH. The structure and properties of the cell surface coat. *Int Rev Cytol.* (1976) 45:291–382. 10.1016/S0074-7696(08)60081-9 60298 PMC8332889

[98] Aird WC. Spatial and temporal dynamics of the endothelium. *J Thromb Haemost.* (2005) 3:1392–406. 10.1111/j.1538-7836.2005.01328.x 15892866

[99] Johansson PI Henriksen HH Stensballe J Gybel-Brask M Cardenas JC Baer LA Traumatic endotheliopathy: a prospective observational study of 424 severely injured patients. *Ann Surg.* (2017) 265:597–603. 10.1097/SLA.0000000000001751 27144442 PMC5300027

[100] Gonzalez Rodriguez E Ostrowski SR Cardenas JC Baer LA Tomasek JS Henriksen HH Syndecan-1: a quantitative marker for the endotheliopathy of trauma. *J Am Coll Surg.* (2017) 225:419–27. 10.1016/j.jamcollsurg.2017.05.012 28579548

[101] Halbgebauer R Braun CK Denk S Mayer B Cinelli P Radermacher P Hemorrhagic shock drives glycocalyx, barrier and organ dysfunction early after polytrauma. *J Crit Care.* (2018) 44:229–37. 10.1016/j.jcrc.2017.11.025 29175047

[102] Richards JE Samet RE Grissom TE. Scratching the surface: endothelial damage in traumatic hemorrhagic shock. *Adv Anesth.* (2021) 39:35–51. 10.1016/j.aan.2021.07.003 34715980

[103] Tiruppathi C Minshall RD Paria BC Vogel SM Malik AB. Role of Ca2+ signaling in the regulation of endothelial permeability. *Vasc Pharm.* (2003) 39:173–85. 10.1016/S1537-1891(03)00007-7 12747958

[104] Reitsma S Slaaf DW Vink H Van Zandvoort MA Oude Egbrink MG. The endothelial glycocalyx: composition, functions, and visualization. *Pflugers Arch.* (2007) 454:345–59. 10.1007/s00424-007-0212-8 17256154 PMC1915585

[105] Chappell D Westphal M Jacob M. The impact of the glycocalyx on microcirculatory oxygen distribution in critical illness. *Curr Opin Anaesthesiol.* (2009) 22:155–62. 10.1097/ACO.0b013e328328d1b6 19307890

[106] Biddle C. Like a slippery fish, a little slime is a good thing: the glycocalyx revealed. *AANA J.* (2013) 81:473–80. 24597010

[107] Aditianingsih D George YWH. Guiding principles of fluid and volume therapy. *Best Pract Res Clin Anaesthesiol.* (2014) 28:249–60. 10.1016/j.bpa.2014.07.002 25208960

[108] Gall LS Vulliamy P Gillespie S Jones TF Pierre RSJ Breukers SE The S100A10 pathway mediates an occult hyperfibrinolytic subtype in trauma patients. *Ann Surg.* (2019) 269:1184–91. 10.1097/SLA.0000000000002733 31082919

[109] Moore EE Moore HB Kornblith LZ Neal MD Hoffman M Mutch NJ Trauma-induced coagulopathy. *Nat Rev Dis Primers.* (2021) 7:30. 10.1038/s41572-021-00264-3 33927200 PMC9107773

[110] Krocker JD Lee KH Henriksen HH Wang Y-WW Schoof EM Karvelsson ST Exploratory investigation of the plasma proteome associated with the endotheliopathy of trauma. *Int J Mol Sci.* (2022) 23:6213. 10.3390/ijms23116213 35682894 PMC9181752

[111] Fu BM Tarbell JM. Mechano-sensing and transduction by endothelial surface glycocalyx: composition, structure, and function. *Wiley Interdiscip Rev Syst Biol Med.* (2013) 5:381–90. 10.1002/wsbm.1211 23401243 PMC4157334

[112] van Hinsbergh VW. Endothelium–role in regulation of coagulation and inflammation. *Semin Immunopathol.* (2012) 34:93–106. 10.1007/s00281-011-0285-5 21845431 PMC3233666

[113] Schott U Solomon C Fries D Bentzer P. The endothelial glycocalyx and its disruption, protection and regeneration: a narrative review. *Scand J Trauma Resusc Emerg Med.* (2016) 24:48. 10.1186/s13049-016-0239-y 27068016 PMC4828893

[114] Hahn RG. Water content of the endothelial glycocalyx layer estimated by volume kinetic analysis. *Intensive Care Med Exp.* (2020) 8:29. 10.1186/s40635-020-00317-z 32651934 PMC7351889

[115] Moore KH Murphy HA George EM. The glycocalyx: a central regulator of vascular function. *Am J Physiol Regul Integr Comp Physiol.* (2021) 320:R508–18. 10.1152/ajpregu.00340.2020 33501896 PMC8238147

[116] Luft JH. Fine structures of capillary and endocapillary layer as revealed by ruthenium red. *Fed Proc.* (1966) 25:1773–83. 5927412

[117] Bazzoni G Dejana E. Endothelial cell-to-cell junctions: molecular organization and role in vascular homeostasis. *Physiol Rev.* (2004) 84:869–901. 10.1152/physrev.00035.2003 15269339

[118] Woodcock TE Woodcock TM. Revised Starling equation and the glycocalyx model of transvascular fluid exchange: an improved paradigm for prescribing intravenous fluid therapy. *Br J Anaesth.* (2012) 108:384–94. 10.1093/bja/aer515 22290457

[119] Chappell D Jacob M. Role of the glycocalyx in fluid management: small things matter. *Best Pract Res Clin Anaesthesiol.* (2014) 28:227–34. 10.1016/j.bpa.2014.06.003 25208958

[120] Brohi K Cohen MJ Ganter MT Matthay MA Mackersie RC Pittet JF. Acute traumatic coagulopathy: initiated by hypoperfusion: modulated through the protein C pathway? *Ann Surg.* (2007) 245:812–8. 10.1097/01.sla.0000256862.79374.31 17457176 PMC1877079

[121] Holcomb JB Jenkins D Rhee P Johannigman J Mahoney PF Mehta S Damage control resuscitation: directly addressing the early coagulopathy of trauma. *J Trauma.* (2007) 62:307–10. 10.1097/TA.0b013e3180324124 17297317

[122] Moore HB Moore EE Gonzalez E Chapman MP Chin TL Silliman CC Hyperfibrinolysis, physiologic fibrinolysis, and fibrinolysis shutdown: the spectrum of postinjury fibrinolysis and relevance to antifibrinolytic therapy. *J Trauma Acute Care Surg.* (2014) 77:811–7. 10.1097/TA.0000000000000341 25051384 PMC4370273

[123] Dobson GP Letson HL Sharma R Sheppard F Cap AP. Mechanisms of early traumatic-induced coagulopathy (TIC): the clot thickens or not? *J Trauma Acute Care Surg.* (2015) 79:301–9. 10.1097/TA.0000000000000729 26218701

[124] D’elia RV Harrison K Oyston PC Lukaszewski RA Clark GC. Targeting the “cytokine storm” for therapeutic benefit. *Clin Vaccine Immunol.* (2013) 20:319–27. 10.1128/CVI.00636-12 23283640 PMC3592351

[125] Corps KN Roth TL McGavern DB. Inflammation and neuroprotection in traumatic brain injury. *JAMA.* (2015) 72:355–62. 10.1001/jamaneurol.2014.3558 25599342 PMC5001842

[126] Mira JC Brakenridge SC Moldawer LL Moore FA. Persistent inflammation, immunosuppression and catabolism syndrome. *Crit Care Clin.* (2017) 33:245–58. 10.1016/j.ccc.2016.12.001 28284293 PMC5351769

[127] Qasim Z Butler FK Holcomb JB Kotora JG Eastridge BJ Brohi K Selective prehospital advanced resuscitative care – developing a strategy to prevent prehospital deaths from noncompressible torso hemorrhage. *Shock.* (2022) 57:7–14. 10.1097/SHK.0000000000001816 34033617

[128] Moore EE Moore FA Harken AH Johnson JL Ciesla D Banerjee A. The two-event construct of postinjury multiple organ failure. *Shock.* (2005) 24(Suppl. 1):71–4. 10.1097/01.shk.0000191336.01036.fe 16374376

[129] Dewar DC Butcher NE King KL Balogh ZJ. Post injury multiple organ failure. *Trauma.* (2011) 13:81–91. 10.1177/1460408610386657

[130] Zeng Y Tarbell JM. The adaptive remodeling of endothelial glycocalyx in response to fluid shear stress. *PLoS One.* (2014) 9:e86249. 10.1371/journal.pone.0086249 24465988 PMC3896483

[131] Hahn RG Patel V Dull RO. Human glycocalyx shedding: systematic review and critical appraisal. *Acta Anaesthesiol Scand.* (2021) 65:590–606. 10.1111/aas.13797 33595101

[132] Dobson GP. On being the right size: heart design, mitochondrial efficiency, and lifespan potential. *Clin Exp Pharm Physiol.* (2003) 30:590–7. 10.1046/j.1440-1681.2003.03876.x 12890185

[133] Kluge MA Fetterman JL Vita JA. Mitochondria and endothelial function. *Circ Res.* (2013) 112:1171–88. 10.1161/CIRCRESAHA.111.300233 23580773 PMC3700369

[134] Bhatti JS Bhatti GK Reddy PH. Mitochondrial dysfunction and oxidative stress in metabolic disorders – a step towards mitochondria based therapeutic strategies. *Biochim Biophys Acta Mol Basis Dis.* (2017) 1863:1066–77. 10.1016/j.bbadis.2016.11.010 27836629 PMC5423868

[135] Berry BJ Trewin AJ Amitrano AM Kim M Wojtovich AP. Use the protonmotive force: mitochondrial uncoupling and reactive oxygen species. *J Mol Biol.* (2018) 430:3873–91. 10.1016/j.jmb.2018.03.025 29626541 PMC6170739

[136] Canyon SJ Dobson GP. The effect of adenosine and lidocaine infusion on myocardial high energy phosphates and pH during regional ischemia in the rat model in vivo. *Can J Physiol Pharmacol.* (2006) 84:903–12. 10.1139/y06-035 17111035

[137] Argaud L Gateau-Roesch O Muntean D Chalabreysse L Loufouat J Robert D Specific inhibition of the mitochondrial permeability transition prevents lethal reperfusion injury. *J Mol Cell Cardiol.* (2005) 38:367–74. 10.1016/j.yjmcc.2004.12.001 15698843

[138] Baines CP. The mitochondrial permeability transition pore as a target of cardioprotective signaling. *Am J Physiol Heart Circ Physiol.* (2007) 293:H903–4. 10.1152/ajpheart.00575.2007 17513492

[139] West AP Shadel GS Ghosh S. Mitochondria in innate immune responses. *Nat Rev Immunol.* (2011) 11:389–402. 10.1038/nri2975 21597473 PMC4281487

[140] Dehne N Brüne B. Sensors, transmitters, and targets in mitochondrial oxygen shortage-a hypoxia-inducible factor relay story. *Antioxid Redox Signal.* (2014) 20:339–52. 10.1089/ars.2012.4776 22794181

[141] Kunkel GH Chaturvedi P Tyagi SC. Mitochondrial pathways to cardiac recovery: TFAM. *Heart Fail Rev.* (2016) 21:499–517. 10.1007/s10741-016-9561-8 27166683 PMC4985491

[142] Cherry AD Piantados CA. Regulation of mitochondrial biogenesis and its intersection with inflammatory responses. *Antioxid Redox Signal.* (2015) 22:965–76. 10.1089/ars.2014.6200 25556935 PMC4390030

[143] Zhao Z Wang M Tian Y Hilton T Salsbery B Zhou EZ Cardiolipin-mediated procoagulant activity of mitochondria contributes to traumatic brain injury–associated coagulopathy in mice. *Blood.* (2016) 127:2763–72. 10.1182/blood-2015-12-688838 27002118 PMC4891956

[144] Cap AP Hunt B. Acute traumatic coagulopathy. *Curr Opin Crit Care.* (2014) 20:638–45. 10.1097/MCC.0000000000000158 25340382

[145] Boudreau LH Duchez AC Cloutier N Soulet D Martin N Bollinger J Platelets release mitochondria serving as substrate for bactericidal group IIA-secreted phospholipase A2 to promote inflammation. *Blood.* (2014) 124:2173–83. 10.1182/blood-2014-05-573543 25082876 PMC4260364

[146] Riedel B Rafat N Browne K Burbury K Schier R. Perioperative implications of vascular endothelial dysfunction: current understanding of this critical sensor-effector organ. *Curr Anesthesiol Rep.* (2013) 3:151–61. 10.1007/s40140-013-0024-7

[147] Johansson PI Stensballe J Ostrowski SR. Shock induced endotheliopathy (SHINE) in acute critical illness - a unifying pathophysiologic mechanism. *Crit Care.* (2017) 21:25. 10.1186/s13054-017-1605-5 28179016 PMC5299749

[148] Henriksen HH Mcgarrity S Sigurethardottir RS Nemkov T D’alessandro A Palsson BO Metabolic systems analysis of shock-induced endotheliopathy (SHINE) in trauma: a new research paradigm. *Ann Surg.* (2020) 272:1140–8. 10.1097/SLA.0000000000003307 31274658

[149] Hart DA. Human heterogeneity and survival of the species: how did it arise and being sustained?—the conundrum facing researchers. *J Biomed Sci Eng.* (2021) 14:212–21. 10.4236/jbise.2021.145018

[150] Tsukamoto T Pepe HC. Animal models for trauma research: what are the options? *Shock.* (2009) 31:3–10. 10.1097/SHK.0b013e31817fdabf 18636048

[151] Dobson GP Letson HL Biros E Morris JL. Specific pathogen-free (SPF) animal status as a variable in biomedical research: have we come full circle? *EBioMedicine (Lancet).* (2019) 41:42–3. 10.1016/j.ebiom.2019.02.038 30803932 PMC6443024

[152] Letson HL Morris JL Biros E Dobson GP. Conventional and specific-pathogen free rats respond differently to anesthesia and surgical trauma. *Sci Rep.* (2019) 9:9399. 10.1038/s41598-019-45871-z 31253875 PMC6599031

[153] Dobson GP Morris J Biros E Letson HL. Specific pathogen-free animals for civilian and military trauma: a cautionary note in the translation of new drug therapies. *Shock.* (2020) 54:232–6. 10.1097/SHK.0000000000001495 32665536

[154] Foster HL. Housing of disease-free vertebrates. *Ann N Y Acad Sci.* (1959) 78:80–8. 10.1111/j.1749-6632.1959.tb53096.x 13824112

[155] Masopust D Sivula CP Jameson SC. Of mice, dirty mice and men: using mice to understand human immunology. *J Immunol.* (2017) 199:383–8. 10.4049/jimmunol.1700453 28696328 PMC5512602

[156] Beura LK Hamilton SE Bi K Schenkel JM Odumade OA Casey KA Normalizing the environment recapitulates adult human immune traits in laboratory mice. *Nature.* (2016) 532:512–6. 10.1038/nature17655 27096360 PMC4871315

[157] Schubert KA Boerema AS Vaanholt LM De Boer SF Strijkstra AM Daan S. Daily torpor in mice: high foraging costs trigger energy-saving hypothermia. *Biol Lett.* (2010) 6:132–5. 10.1098/rsbl.2009.0569 19710051 PMC2817250

[158] Dobson GP. The August Krogh principle: seeking unity in diversity. *Shock.* (2014) 42:480. 10.1097/SHK.0000000000000229 25320913

[159] Bouma HR Carey HV Kroese GM. Hibernation: the immune system at rest? *J Leukocyte Biol.* (2010) 88:619–24. 10.1189/jlb.0310174 20519639

[160] Franconi F Campesi I Colombo D Antonini P. Sex-gender variable: methodological recommendations for increasing scientific value of clinical studies. *Cells.* (2019) 8:476. 10.3390/cells8050476 31109006 PMC6562815

[161] Gölz C Kirchhoff FP Westerhorstmann J Schmidt M Hirnet T Rune GM Sex hormones modulate pathogenic processes in experimental traumatic brain injury. *J Neurochem.* (2019). [Epub ahead of print]. 10.1111/jnc.14678 30790293

[162] Gupte R Brooks WM Vukas RR Pierce JD Harris JL. Sex differences in traumatic brain injury: what we know and what we should know. *J Neurotrauma.* (2019). [Epub ahead of print]. 10.1089/neu.2018.6171 30794028 PMC6818488

[163] Choudhry MA Bland KI Chaudry IH. Trauma and immune response–effect of gender differences. *Injury.* (2007) 38:1382–91. 10.1016/j.injury.2007.09.027 18048037 PMC2692838

[164] Chaudry IH Bland KI. Cellular mechanisms of injury after major trauma. *Br J Surg.* (2009) 96:1097–8. 10.1002/bjs.6697 19787761 PMC2947352

[165] Shoemaker WC Beez M. Pathophysiology, monitoring, and therapy of shock with organ failure. *Appl Cardiopul Pathophysiol.* (2010) 14: 5–15.

[166] Dinarello CA. Overview of the IL-1 family in innate inflammation and acquired immunity. *Immunol Rev.* (2018) 281:8–27. 10.1111/imr.12621 29247995 PMC5756628

[167] Mehta P Mcauley DF Brown M Sanchez E Tattersall RS Mansen JJ. COVID-19: consider cytokine storm syndromes and immunosuppression. *Lancet.* (2020) 395:1033–4. 10.1016/S0140-6736(20)30628-0 32192578 PMC7270045

[168] Moore TJ Zhang H Anderson G Alexander GC. Estimated costs of pivotal trials for novel therapeutic agents approved by the US food and drug administration, 2015-2016. *JAMA Intern Med.* (2018) 178:1451–7. 10.1001/jamainternmed.2018.3931 30264133 PMC6248200

[169] Moore FD. The growth of surgical biology. *Ann Surg.* (1953) 138:807–22. 10.1097/00000658-195311000-00023 13092819 PMC1609390

[170] Letson HL Granfeldt A Jensen TH Mattson TH Dobson GP. ALM supports a high flow, hypotensive, vasodilatory state with improved O2 delivery and cerebral protection in a pig model of non-compressible hemorrhage. *J Surg Res.* (2020) 253:127–38. 10.1016/j.jss.2020.03.048 32353638

[171] Letson HL Dobson GP. 3.0% NaCl adenosine, lidocaine, Mg2+ (ALM) bolus and 4 hours ‘drip’ infusion reduces non-compressible hemorrhage by 60% in a rat model. *J Trauma Acute Care Surg.* (2017) 82:1063–72. 10.1097/TA.0000000000001454 28520687

[172] Letson HL Dobson GP. Adenosine, lidocaine and Mg2+ (ALM) fluid therapy attenuates systemic inflammation, platelet dysfunction and coagulopathy after non-compressible truncal hemorrhage. *PLos One.* (2017) 12:e0188144. 10.1371/journal.pone.0188144 29145467 PMC5690633

[173] Letson HL Dobson GP. Adenosine, lidocaine and Mg2+ (ALM) resuscitation fluid protects against experimental traumatic brain injury. *J Trauma Acute Care Surg.* (2018) 84:908–16. 10.1097/TA.0000000000001874 29554045

[174] Letson HL Morris JL Biros E Dobson GP. ALM fluid therapy leads to 72 hr survival after hemorrhagic shock: a model for studying differential gene expression and extending biological time. *J Trauma Acute Care Surg.* (2019) 87:606–13. 10.1097/TA.0000000000002397 31162330

[175] Griffin MJ Letson HL Dobson GP. Adenosine, lidocaine and Mg2+ (ALM) induces a reversible hypotensive state, reduces lung edema and prevents coagulopathy in the rat model of polymicrobial sepsis. *J Trauma Acute Care Surg.* (2014) 77:471–8. 10.1097/TA.0000000000000361 25159253

[176] Griffin MJ Letson HL Dobson GP. Small-volume adenosine, lidocaine and Mg2+ (ALM) 4 hour infusion leads to 88% survival after 6 days of experimental sepsis in the rat without antibiotics. *Clin Vaccine Immunol.* (2016) 23:863–72. 10.1128/CVI.00390-16 27581435 PMC5098019

[177] Granfeldt A Letson HL Dobson GP Shi W Vinten-Johansen J Tonnesen E. Cardioprotective and anti-inflammatory effects of treatment with adenosine, lidocaine and Mg2+ in a porcine model of endotoxemia. *Circulation.* (2013) 18:682–70. 10.1186/s13054-014-0682-y PMC4301798 25497775

[178] Letson HL Biros E Morris JL Dobson GP. ALM fluid therapy shifts sympathetic hyperactivity to parasympathetic dominance in the rat model of non-compressible hemorrhagic shock. *Shock.* (2022) 57:264–73. 10.1097/SHK.0000000000001886 34798632

[179] Letson HL Dobson GP. Differential contributions of platelets and fibrinogen to early coagulopathy in a rat model of hemorrhagic shock. *Thromb Res.* (2016) 141:58–61. 10.1016/j.thromres.2016.03.007 26970714

[180] Torres Filho IP Torres LN Salgado C Dubick MA. Novel adjunct drugs reverse endothelial glycocalyx damage after hemorrhagic shock in rats. *Shock.* (2017) 48:583–9. 10.1097/SHK.0000000000000895 28489728

[181] Seyhan AA. Lost in translation: the valley of death across preclinical and clinical divide – identification of problems and overcoming obstacles. *Transl Med Commun.* (2019) 4:18. 10.1186/s41231-019-0050-7

[182] Downing NS Shah ND Aminawung JA Pease AM Zeitoun JD Krumholz HM Postmarket safety events among novel therapeutics approved by the US food and drug administration between 2001 and 2010. *JAMA.* (2017) 317:1854–63. 10.1001/jama.2017.5150 28492899 PMC5815036

[183] Huston JM Tracey KJ. The pulse of inflammation: heart rate variability, the cholinergic anti-inflammatory pathway and implications for therapy. *J Intern Med.* (2011) 269:45–53. 10.1111/j.1365-2796.2010.02321.x 21158977 PMC4527046

[184] Matteoli G Boeckxstaens GE. The vagal innervation of the gut and immune homeostasis. *Gut.* (2013) 62:1214–22. 10.1136/gutjnl-2012-302550 23023166 PMC3711371

[185] Dobson GP. Addressing the global burden of sepsis: importance of a systems-based approach. *Crit Care Med.* (2014) 42:e797–8. 10.1097/CCM.0000000000000595 25402302

[186] Chappell D Jacob M Hofmann-Kiefer K Bruegger D Rehm M Conzen P Hydrocortisone preserves the vascular barrier by protecting the endothelial glycocalyx. *Anesthesiology.* (2007) 107:776–84. 10.1097/01.anes.0000286984.39328.96 18073553

[187] Pries AR Secomb TW Gaehtgens P. The endothelial surface layer. *Pflügers Arch.* (2000) 440:653–66. 10.1007/s004240000307 11007304

[188] Pillinger NL Kam PCA. Endothelial glycocalyx: basic science and clinical implications. *Anaesth Intensive Care.* (2017) 45:3. 10.1177/0310057X1704500305 28486888

[189] Vink H Duling BR. Identification of distinct luminal domains for macromolecules, erythrocytes, and leukocytes within mammalian capillaries. *Circ Res.* (1996) 79:581–9. 10.1161/01.RES.79.3.581 8781491

[190] Squire JM Chew M Nneji G Neal C Barry J Michel C. Quasi-periodic substructure in the microvessel endothelial glycocalyx: a possible explanation for molecular filtering? *J Struct Biol.* (2001) 136:239–55. 10.1006/jsbi.2002.4441 12051903

[191] Weinbaum S Zhang X Han Y Vink H Cowin SC. Mechanotransduction and flow across the endothelial glycocalyx. *Proc Natl Acad Sci USA.* (2003) 100:7988–95. 10.1073/pnas.1332808100 12810946 PMC164700

[192] Arkill KP Knupp C Michel CC Neal CR Qvortrup K Rostgaard J Similar endothelial glycocalyx structures in microvessels from a range of mammalian tissues: evidence for a common filtering mechanism? *Biophys J.* (2011) 101:1046–56. 10.1016/j.bpj.2011.07.036 21889441 PMC3164174

[193] Arkill KP. A reinterpretation of evidence for the endothelial glycocalyx filtration structure. *Front Cell Dev Biol.* (2021) 9:734661. 10.3389/fcell.2021.734661 34540847 PMC8442954

